# Baunscheidtismus

**Published:** 1861-09

**Authors:** 


### Editors' Table.

Baunscheidtismus.—In our last editorial table-talk we brought up a picture of insanity, painted by the master-hand of genius—the Life of Don Quixote, the renowned Knight of La Mancha, by the inimitable Cervantes. On the present occasion we are required, it must be as a penance for previous shortcomings and omissions, to say something about a cloudy daub, which has neither life nor fancy to recommend it, nor even the negative merit of unintentional caricature, to render it amusing. To be lost in a thick fog, with nothing but a will-of-the-wisp to guide us, to try and steer by a compass which will not traverse, or to listen to the insane clutterings of an idiot, are no small hardships in their way; but they sink into insignificance compared with the punishment of being obliged to read through a small volume now on our table, entitled "*Baunscheidtismus*, by the Inventor of the New Curing Method, Charles Baunscheidt."* The dream-land of German fancy pleases while it mystifies, German lore commands our respect, and its conjectural criticism, even when it runs into the transcendental, is ever suggestive, if not directly instructive; but, woe to us, there is neither fancy, nor learning, nor criticism, any more than there is philosophy or pertinent fact, in the present work. In vain shall we look for any reasoning in pages which abound in commonplaces and platitudes, joined together without method, and unenlivened by a single guiding principle in medical science. We are continually reminded of an ignorant man who would make some show of learning, of a pretender to inventive genius who is essentially and incurably stupid, and of a pseudo-philanthropist who is intent on replenishing his purse by taxing the credulity of his fellow-men. In this last device, Baunscheidt has, we learn, been quite successful, and has already become possessor of a large fortune.

Much as we have ever reprobated and despised quacks and quackery, we cannot but acknowledge that the criminals may plead strong temptation to palliate their nefarious deeds. Among these are the readiness and almost eagerness of people of all classes to be duped, and the pleasure which they who deceive must experience at the success of their efforts, as implying a mental superiority for the time being. A quack is, continually, carrying on a hoax on a large scale: he will sometimes succeed in victimizing a whole community, while exciting also its wonderment and eliciting its applause: he is, for the nonce, the adroit juggler, laughing with and at his admirers, who willingly replenish

* First English edition, translated from the sixth original edition. By John Cheyne and L. Haymann. Bonn.

his purse, in return for his having deluded them. There is an innate love of the marvelous in mankind, and, provided this love can be gratified, they will not criticise very closely the selection of means, even though these should border on imposture. The quack may, therefore, be sure of the ready sympathy from the crowd, whenever he makes boasted promises of possessing a panacea or an elixir of life. The more extravagant his pretensions the greater their belief. He is also linked to the crowd by a community of ignorance, general and special—general, in all that relates to science and the rules of logic—special, in all that relates to the structure, functions, and diseases of the human body, and of the successive and alternate use of remedies. Hence it follows, as a result of this sympathy, that an exposure of the ignorance, illiterateness, deception, and mendacity of a quack by an experienced and well-read physician, is not only coldly received by the multitude, but is regarded by many in the light of persecution. We wish that we could find an excuse of ignorance in extenuation of those who are so ready, on the slightest grounds, and often without any grounds at all, to give their certificates in favor of the last quack remedy. Ignorant they doubtless are of the subject—the nature of the disease, and the circumstances under which it can be most readily and safely arrested; but as clergymen, lawyers, and litterateurs, they will not be disposed to plead ignorance, in bar of judgment, for encouraging fraudulent pretensions to cure diseases which are incurable, or other diseases, by means which are not in reality employed. The radical defect in the minds of these heedless certifiers is the want of a matured system of ethics, on the one hand, and of the habit of logical reasoning on the other. A little knowledge of the laws of evidence would make them, in an intellectual point of view, refuse at once to lend their names in attestation of a thing which has no support from the first elements of these laws, as it certainly would, ethically considered, make them shrink from encouraging that which bears on its face the stamp of imposture.

If ignorance of the law excuseth no man, so neither ought merely good intentions to excuse a man of average intelligence and education from certifying to the truth of that which he has not taken pains to examine and scrutinize, by the aid of all the lights at his disposal. A good-natured yieldingness to oblige a needy, forward, and hypocritical applicant, by our lending him our names, will be a small offset against the injury to the health and pockets of a large number of credulous persons, who are induced, mainly on the strength of our certificates, to purchase the nostrums of the said quack. But it may be asked, are people injured by this means? This is the question which every thoughtful and conscientious man will ask himself, after he has solved, to his own satisfaction, the other question, viz., whether the articles which the quack asserts that he employs do really enter into the composition of his nostrum? and if so, whether said articles are not inert or powerless, as ascertained by the ample experience of medical men? and again, whether the same substance or compound can be a cure-all, adapted to diseases of the most various and opposite characters? Even if, without entering into a rigid scrutiny of the merits of the particular nostrum, a person would reflect for a moment, that as the whole history of quackery shows it to be an uninterrupted series of fraud and imposture, so must there be a violent presumption that every new exhibition of it is also false and fraudulent. A single pertinent fact should convince the most determined supporter of quackery. It is, that no one of the hundreds of cure-alls continues in use and vogue beyond the lifetime of its proprietor and putative inventor.

We have touched on the palliation of quackery by the strong temptations to engage in it with a prospect of success. That which many regard as a valid plea in favor of the quack, or at least in abatement of public censure, if not of punishment, does not impress us with any weight. We allude now to poverty. Were such a plea admissible, law and ethics would have to wink at the offense of the sturdy beggar, who steals a loaf to allay the pangs of hunger; of the forger, who is impelled to extricate himself from pecuniary embarrassment, which will, if not relieved, be followed by the destitution of his family; and of a pickpocket, urged to rob by similar considerations.

Thus much on quacks and quackery in general. We now begin our ungracious task of noticing an individual exhibition in the person of Charles Baunscheidt, and in his book, Baunscheidtismus. This inventor of the Lebenswecker and reformer of medical doctrine and practice, like nearly all others of his class, began his career without any previous training, and in entire ignorance of the subjects of life and disease, which all at once, moved thereto, it must be, by a sudden fit of inspiration, he discusses in the most bold and confident manner. Possessed of considerable mechanical ingenuity, he first came before the public as the inventor of "a proof weapon" and a new helmet, which were adopted in the Prussian army; and next he directed his attention to the construction and perfecting of plowing machines. The prevalence of a destructive epidemic disease among horned cattle led him to meditate "on the homœopathic principles of Hahnemann, which pleased him, and a thousand different experiments upon animals proved to him that it deserved to be preferred to the violent allopathy." The nature and scope of these experiments are not mentioned.

"Later on, he wrote a book on splenitis (fatal distemper of cattle), wherein he advocated the treatment of horned cattle on the principles of Hahnemann." This do-nothing treatment was, very likely, preferable to the crude practices of ignorant cow-doctors. With so restless a mind as that of Baunscheidt, the transition from treating the animals of the bovine class to undertaking the cure of the members of the genus *homo* was easily made; and behold him now a medical man, ready, of course, for any and all vagaries of doctrine and practice, now that he had been smitten with homœopathy. The immediate incitement to this course was his grief at the death of a friend, who fell a victim to a lingering disease—name or nature not stated. The sympathizing Baunscheidt kept up the spirits of his friend by such words as the following, in reply to a remark of the sufferer, that physic could afford him no relief: "They [the physicians] have half killed you by medicaments, my dear friend!" Then follows a rhapsody, in the jargon which pervades nearly every page of the book, and in which sense and grammar are alike forgotten. "It is a misdemeanor crying to heaven, when the medical faculty have made it a practice for the last thousand years with the sanctuary of human life; that must be changed, if one could only induce everybody to study, and learn to know the connection of the single parts of his own body, then every man would become the best physician for himself, become a free agent, and thus would the old medical science fall away, and at length sink into utter oblivion." It did not occur to this would-be reformer, that, for many hundreds of years past, one of the divisions of medical science consisted in a study and knowledge of the single parts of the human body. The field of anatomy was to him a *terra incognita*. He must have thought that he was the first to enter on it; for, when he began dissecting sheep, swine, dogs, and cats, and when he made excursions over mountain and valley, and studied "in the woods and in the fields the botany of indigenous plants," he seems not to have been aware that others had collected information on these points before him. How long Baunscheidt would have continued in this course of observation and study cannot be conjectured; but it had its termination in one of those lucky incidents or chances which occur so often in the history of genius. His biographer, who is continually straining after effect by magniloquent phrases and odd analogies, ought to have told us that, as the theory of gravitation was suggested to Newton by the fall of an apple from the tree, and that of the oscillation of the pendulum to Galileo by the swinging of a lamp suspended from the ceiling of the cathedral at Pisa, so was the idea of the Lebenswecker—the life reviver and restorer, the curer of all ills—suggested to the philosopher Baunscheidt by a swarm of large gnats lighting on a gouty swelling of his left hand, which had become ulcerous, and leaving behind them innumerable marks of their stings. But "how brightened with joy was the eye of the examining thinker, when, a few seconds afterward, he perceived that the whole of the pain in his hand had passed away through the openings made by the stinging probosces of the gnats! and Baunscheidt, as he had formerly said that matters in the materia medica ought to be changed, now cried out exultingly, 'they must be changed, and I have found out the way *how* to do it!' Thus this accidental circumstance," continues the biographer, "served him as a basis whereon to found the invention of a mechanical instrument which is calculated at all times to apply artificially the salutary stings of the gnats, and by such means to awaken again into life the benumbed limbs. Baunscheidt invented his Lebenswecker and his famous 'oleum Baunscheidtii'—the latter supplying the kind of liquid left in the wounds made by the little piercers of the gnats, which convey the matter into the channels of the rheumatic substances."

The modesty, logic, pathology, and therapeutics evinced in the following can scarcely be equaled:—

"So easy as it is to be ill so easy must it be to become well; provided old age, with its natural weakness, is not operating against it. Wherever a system exists which is not able to cure a man under fifty years of age, and being still in the vigor of his life, it is not a life-healing method, and it is of no value. Rheumatism and gout are," he says, "the mothers of almost all other diseases. The remedy which overcomes these diseases is, therefore, their master, and that remedy I have found out."—Q. E. D. Another Euclid, exclaiming "Eureka!"

The "Introduction" to this precious volume treats of "life and its design," in a mystic vein, in which truisms are disguised and philosophy is in harlequinade. In the section headed "What is life?" we read, as part of a luminous answer, the following: "If the homogeneous original structure of the body be supposed the first agent, and the specific substance as the second, we have then the third substrate member, continual movement. May not this be identical with the primeval cause of life?" A fine specimen this, truly, of the dim-obscure. A little farther on we are enlivened by the paradox contained in another question: "Would it be censurable to conclude that prussic acid in its intensity, and possessing the specific power of destroying life, contains the embryo of life's fluid?" The answer must be made by the initiated in Baunscheidtismus. To them, also, as alone able to appreciate their contents, we must leave the sections on "pure spiritual life" and "spiritual physical life." The section on "physical life" gives the gratifying assurance that "life can be no other than the primeval power or the fundamental structure of our being." Speaking of the "Aims of Life," there peeps out from a thick fog this luminous proposition: "that the primeval aim of human life is, at first, no other than the right to life." In an ordinary postulate, a claim and a right are associated—an aim looks to its attainment irrespective of prior right and regardless of attendant wrong.

The author begins his section "on the remedies to preserve life" by a few cursory remarks on the neglect of physical education, in seeming ignorance that the subject has been treated, again and again, with a hundredfold more force and point than he imparts to it. Passing to the question, of the cure of diseases, the author, assuming his own ignorance as the representative of that of educated medical men, advises them, as matters "which the physicians have not known," "to study the system of our own bodies and the countless bodies that surround us, and to which we have a relative position." This self-made doctor and suddenly-illuminated medical philosopher shows his persistent ignorance of the fact that physicians had ever studied anatomy; and that the operation on the human frame of the "circumfusa" and other material agents occupied their attention from the time when Hippocrates wrote his great treatise on Air, Water, and Places, down to the present day, when we have so many capital treatises and reports on both public and private hygiene.

After many pages of pointless assertions, heavy dogmatism, and muddled commonplaces, the great reformer condescends to

"give a comprehensive description of the manipulating and medically operating power of an instrument that has gone forth into the world under the characteristic name of the 'Lebenswecker,' which declares its name and high importance, and which manifestly declares war against most if not all of the old and venerable apothecary's boxes. But what does this singular instrument consist of? Nothing more than of fine-pointed needles fixed in a round plate of steel. These produce artificial pores on the epidermis by an almost painless operation, by which all virulent matters noxious to health, and which lay in different portions of the body, are gradually removed by volatilization, it must be evident to every impartial and reflecting mind that if, instead of applying pernicious medicines *internally*, working their dark and mysterious ways in the body, we can *externally near the seat of the disease*, relieve the suffering patient by means of the Lebenswecker, we maintain that we have fulfilled our long-sought panacea."*

* The italics are in the text.

Whether the running of sentences into each other, in the above extract, has been the work of the author, or is to be credited to the translator, who is evidently ignorant of the grammar and construction of his own vernacular, we do not pretend to say. We have given the text as we find it, but must be excused from inflicting on ourselves and our readers the transcription of the notes by which it is accompanied.

Unable to fix his attention on the few main points which have any show of practical bearing, Baunscheidt could not finish this description of his wonderful instrument—wonderful, in his mind, by its simplicity and the astonishing effects alleged to result from its use—without a long intervening chapter headed "Practical Instruction of the Oleum Baunscheidtii." After this comes a chapter headed "More Precise Description of the Lebenswecker and its Manipulation," with drawings. The complete instrument is represented to consist of an ebony or horn box or tube, the upper part of which being unscrewed, reveals a number of needles fixed on a metallic plate. This plate is attached to a brass spiral spring beneath, which, when the instrument is used, is drawn up into the tube by a handle to the extent of one and a half to two inches. The release of the handle causes a recoil of the spring, and a consequent projection of the needles and their penetration of the skin, to which the mouth of the tube had been previously applied.

The application of the Lebenswecker must be followed by that of the oil of Baunscheidt, (*oleum Baunscheidtii,*) which is all-important for the bringing about of a cure. The inventor of this still secret compound professes to imitate in it the fluid that runs from the gnat, which he declares to be "very salutiferous for the body, because it produces an irritation of the skin, which tends to remove efficaciously and promptly all infectious diseases." If there had been any lurking doubt of the virtues of the oil, and the good sense and integrity of the inventor, it cannot fail to be dispelled by the averment, that this oil "serves to keep together the galvanic juncture of the polary needles, and being a destructor of rust, is, consequently, an especial conservator of them." Every part of the skin touched by the Lebenswecker is to be well anointed with the oil, by means of a feather or a camel's-hair brush. There ensues, on this application, an eruption similar to millet-seed, the size and extent of which vary "according to the quantity of unsound matter in the body; the skin afterward, in a very short time, assumes a warm and healthy red color, and is very expansive and pliable: the patient, after feeling a crawling sensation in his skin, perceives a more general activity in the whole body, and seems in a certain degree transported to a warmer climate, and thus all the body becomes sound and healthy." What a grand termination to so small a beginning!

We next have what is called "Universal and Practical Instruction," which opens with the following aphorism, a true specimen of the enlightened dogmatism of Baunscheidtismus:

"1. Because every dangerous disease has its principal seat in the back, it is agreeable to nature to operate there first, to deliver life from its morbid pressure, along the vertebral column of the spine, in the left and right of it, (see the copper-plate)."

How grateful the reader of this sage direction must be at the careful iteration, "the vertebral column of the spine," so that there can be no mistake as to the precise part in which life is imprisoned, and from which it is to be set free. The patient is cautioned to avoid, during the first three days after the application of the Lebenswecker, all draughts of air and moisture: "The morning ablutions should occupy only a very short time; he should not cleanse any vegetables, and should avoid cellars and other damp places." Our readers must not ask us to tell them what is meant by the above cleansing. No change in the diet is required, except the avoidance of the use of acids.

The copper-plate referred to above represents the nude forms of Adonis and Venus united in a most affectionate embrace; one-half of the anterior surface of the body of the former and the entire back view of the latter being exhibited. The visible parts of the skin of each of these personages is freely spotted, as if just marked with the needles of the instrument, or rather with the pustules of small-pox. The more favored regions are those of the clavicle, the chest near the shoulder, the right hypochondrium and abdomen, the inside of the forearm and calf of the leg, on Adonis; and the back on each side of the spine, more especially in the upper or humeral region, and at the lower end, "near which lies the seat of hemorrhoidal diseases," also over the hip-joint and the calves of the legs, on Venus. This picture, at first glance, might be thought to suggest erotic ideas, but any feeling of this nature must be promptly extinguished by the seemingly eruptive disease of these personages; and, if we adopt the view of Baunscheidt, by the thought of crude humors oozing out from the fair skins of Love and Beauty. Happily, we have no female readers, else we should tremble for the shock to their sentimental loves for the next six months, caused by this mere allusion. How will it be with those who read the book itself?

We would apologize to our readers for occupying any more of their time by quoting the following precious sample of Baunscheidtismus, were it not to furnish additional and conclusive proof of the justness of our strictures, harsh as they may seem, on the impudent pretensions of the new teacher and would-be reformer:—

"Inflammation. The character of inflammation consists in a state of the bodies of blood in the capillary vessels.* Brücke explains the cause by the straitening of the arteries. The incentive which calls forth the inflammation is urging on the fibres of the artery to a spasmodic contraction, by these means entering sluggishly, and ultimately a stoppage takes place, and as the interior diameter of the vessels must become less, and the small bodies have no longer a free current, every mutation is capable of causing only a reaction by the nerves; that the mutation calls forth a neuralgic congestion of the nerves, by which the contracted fibre in the artery is urged on to the spasmodic contraction; the healing process must, therefore, at first, be a compensation of the nervous matter—a removing of the neuralgia—then the vessel's natural diameter is restored, the circulation becomes free, and the inflammation is removed. We apply, also, incentives which are calculated to dissolve the imflammation. Particularly important is this method upon the noble organs, such as the lungs, the brain, the openings in the stomach and the intestines are attacked."

In the chapter already referred to, the title of which is expressed in such original English, "Radical Instruction of the Oleum Baunscheidtii," the author gives a long enumeration of the

* "Capillary or hair vessels are called in anatomy the finest vessels, but are formed for passing *over* the arteries into the nerves."

diseases cured by the instrument and the oil. The most serious and intractable are dismissed in as brief and summary a style, and their cure announced with as much confidence, as the slightest disorders. It must be consoling for us to learn that yellow fever, which the new-light German pathology puts in the same line with jaundice, can be quickly and radically treated by the Lebenswecker—the instrument being applied "strongly on the back, and on the whole surface of the stomach and belly, and particularly on the right side near the liver." The doctrine of sympathetic irritation of other organs with the gastro-intestinal, as taught by Broussais, is pushed *à l'outrance* in the present work. Thus we read :—

> "Affections of the uvula, hoarseness, and chronic diseased windpipe have their seat of disease in the bowels, and the Lebenswecker, applied on the back and on the right and left side of the neck, will have the desired effect."

One would hardly expect such a deduction from the premises. To this family belong diseases new to the American physician, viz.: Inflammation and Consumption of Adam's Apple. They are treated like the former, and of course with the same success. That persistent and most disagreeable squatter in the human body, the tapeworm, is compelled to leave in disgust by the Baunscheidt system. The lockjaw, in tetanus, is cured by the same means, with equal readiness: and so on through the nosological catalogue.

Baunscheidt will not admit of any analogy of his fashion of acupuncture to that in previous use, nor to scarification by lancets, as in cupping; nor must the eruption caused by his ointment be compared for a moment to that following the application of tartar emetic or other means of causing an artificial cutaneous irritation. Small-pox is to be prevented by substituting, in all cases, "the cure of the Lebenswecker" for inoculation. Vaccination is proclaimed to have increased scrofulous complaints in a fearful manner, and given rise both to scrofula and syphilis. After speaking of the pustular eruption on the udder of cows being caused by the "grease" of horses, our sage reformer asks: "Now can any rational person suppose that the venom of a scabbed animal possesses any healing properties?" He talks on the subject of vaccination like a denizen of Sleepy-hollow, whose senses and intellect have been torpid for the last fifty years.

"The Lebenswecker a Prolonger of Life," is the heading of a chapter. Were we obliged to follow any further the author in his garrulities and inanities, our own life would certainly be shortened; and, to prevent this, and anticipating also the exclamation of our readers, *Ohe jam satis!* we close his book with gratitude for the relief thus obtained, and a firm resolve to tear ourselves away from such enforced company at any future time.

